# Implementation of artificial intelligence models in magnetic resonance imaging with focus on diagnosis of rheumatoid arthritis and axial spondyloarthritis: narrative review

**DOI:** 10.3389/fmed.2023.1280266

**Published:** 2023-12-20

**Authors:** Andreea-Iulia Nicoara, Lorena-Mihaela Sas, Cristina Elena Bita, Stefan Cristian Dinescu, Florentin Ananu Vreju

**Affiliations:** ^1^University of Medicine and Pharmacy of Craiova, Craiova, Romania; ^2^Radiology and Medical Imaging Laboratory, Craiova Emergency County Clinical Hospital, Craiova, Romania; ^3^Department of Human Anatomy, University of Medicine and Pharmacy of Craiova, Craiova, Romania; ^4^Department of Rheumatology, University of Medicine and Pharmacy of Craiova, Craiova, Romania

**Keywords:** artificial intelligence, machine learning, deep learning, magnetic resonance imaging, rheumatoid arthritis, axial spondyloarthritis

## Abstract

Early diagnosis in rheumatoid arthritis (RA) and axial spondyloarthritis (axSpA) is essential to initiate timely interventions, such as medication and lifestyle changes, preventing irreversible joint damage, reducing symptoms, and improving long-term outcomes for patients. Since magnetic resonance imaging (MRI) of the wrist and hand, in case of RA and MRI of the sacroiliac joints (SIJ) in case of axSpA can identify inflammation before it is clinically discernible, this modality may be crucial for early diagnosis. Artificial intelligence (AI) techniques, together with machine learning (ML) and deep learning (DL) have quickly evolved in the medical field, having an important role in improving diagnosis, prognosis, in evaluating the effectiveness of treatment and monitoring the activity of rheumatic diseases through MRI. The improvements of AI techniques in the last years regarding imaging interpretation have demonstrated that a computer-based analysis can equal and even exceed the human eye. The studies in the field of AI have investigated how specific algorithms could distinguish between tissues, diagnose rheumatic pathology and grade different signs of early inflammation, all of them being crucial for tracking disease activity. The aim of this paper is to highlight the implementation of AI models in MRI with focus on diagnosis of RA and axSpA through a literature review.

## 1 Introduction

RA is an autoimmune and inflammatory condition that mainly affects the small joints of the hands and can cause a high degree of disability. It is associated with high morbidity and increased socioeconomic burden (1, 2).

Joint inflammation and pain are the main causes that lead to deterioration of the quality of life in the first years, the loss of cartilage and the intraarticular space, together with joint deformities and later ankylosis representing the cause of disability in advanced disease (1).

Diagnosing RA involves a comprehensive assessment, including clinical, laboratory, and imaging findings. Blood tests are often performed to check for the presence of rheumatoid factor (RF) and anti-citrullinated protein antibodies (ACPAs). While the presence of these antibodies can support a diagnosis of RA, their absence does not rule it out. Also, elevated levels of C-reactive Protein (CRP) and Erythrocyte Sedimentation Rate (ESR) indicate the presence of inflammation, which is common in RA.

Thus, early diagnosis and treatment are essential to effectively manage RA and prevent joint damage. The “treat-to-target” (T2T) strategy is an approach used in the management of RA to optimize treatment outcomes by setting specific treatment targets and adjusting therapy based on regular assessments of disease activity. The primary goal is to achieve and maintain remission or low disease activity, thereby preventing joint damage and improving long-term outcomes for individuals with RA. Nowadays, imaging plays an important role in both diagnostic and management of RA (3).

AxSpA is a type of inflammatory arthritis that primarily affects the spine and SIJ. There are two subtypes of axSpA: non-radiographic axSpA (nr-axSpA) and axSpA (axSpA). There are some common features associated with axSpA: the presence of the human leukocyte antigen B27 (HLA-B27), the presence of inflammatory back pain, the inflammation of the spine and SIJ, the inflammation at the sites where ligaments and tendons attach to the bone, known as entheses, and the higher prevalence in young male patients (4, 5).

If there is a high clinical suspicion of axSpA and the conventional radiography is normal, the basic procedure for diagnosing this pathology is to perform a MRI of SIJ, this being the most accurate imaging method for diagnosing sacroiliitis. The most sensitive method that allows the highlighting of bone marrow edema (BME) and periarticular inflammation is MRI, compared to low dose computed tomography (CT) that only allows the identification of subchondral bone erosions and ankylosis (6, 7).

Thus, early diagnostic and treatment of axSpA are crucial in managing symptoms and preventing long-term complications. The treatment aims to alleviate symptoms, reduce inflammation, maintain function, and improve the overall quality of life for individuals affected by the condition. The approach to treatment can involve a combination of medications, physical therapy, and lifestyle modifications. For individuals with more severe or persistent symptoms, the biologic and targeted disease-modifying anti-rheumatic drugs (DMARDs) are able to prevent the loss of functional capacity, if administered before to the structural lesions (4, 5, 8, 9).

**Figure 1:**
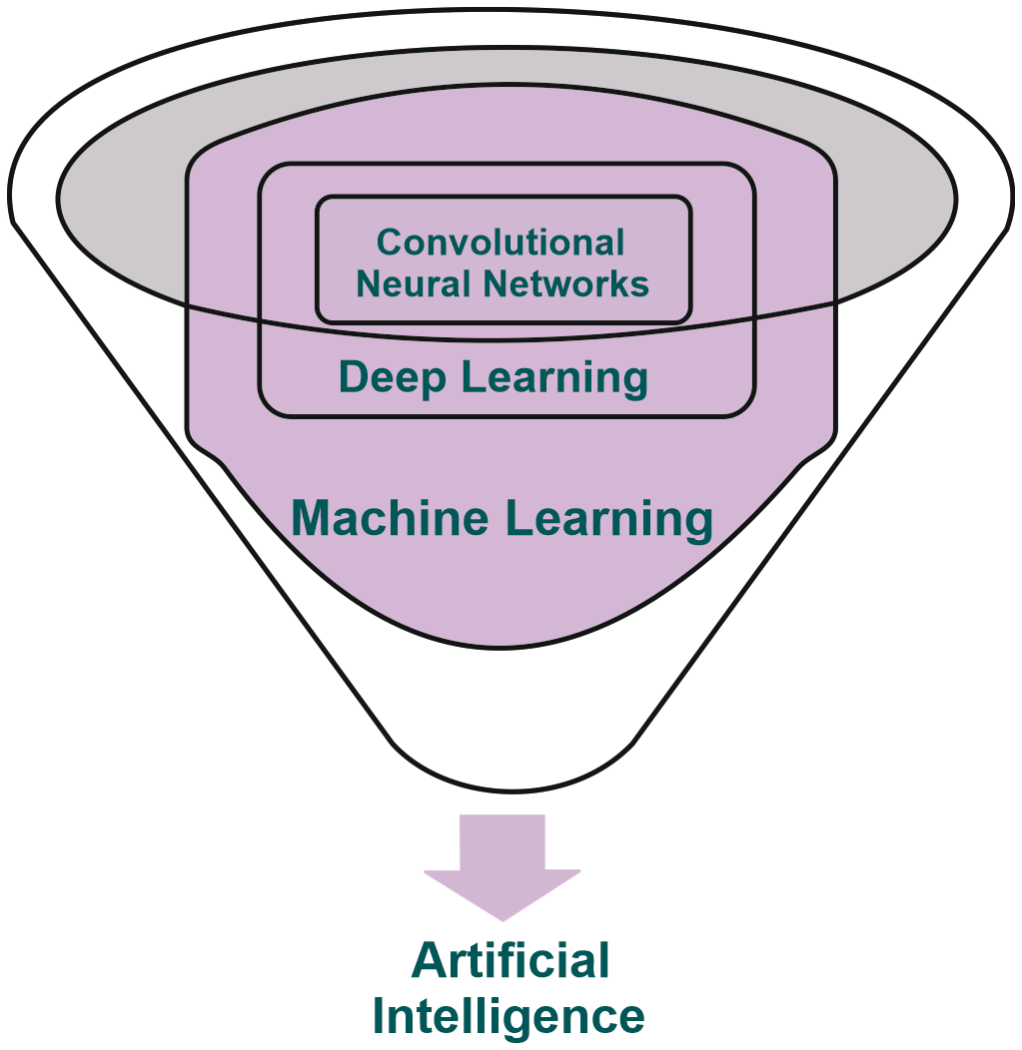
Illustrative depiction showcasing the interconnection among artificial intelligence, machine learning, deep learning, and convolutional neural networks.

AI is a branch of computer science with the capability to emulate human intelligence (10, 11), which has been successfully implemented in the field of medical imaging in the past few decades. ML is one of the main subdivisions of AI and which relies on the use of algorithms for processing input data and aid the physician in various clinical setting (12). DL, a subset of ML, is based on the utilization of artificial neural networks (ANN) with the capacity to address complex requirements and work with large data sets with limited need for human intervention. It can address complex requirements without the need for human brain intervention. This subdivision has experienced substantial growth in recent years, standing as the primary area of interest within the field of AI and is set to undergo continuous evolution (13–17). The notable development of Convolutional Neural Networks (CNNs) within DL has led to significant achievements in the field of imaging by efficiently addressing problems based on image acquisitions (14, 18–20) (Figure 1). CNNs are neural networks trained to receive various input data, for example imaging scans, and are subsequently capable of classifying them based on the received features (output data) (18, 21).

**Figure 2:**
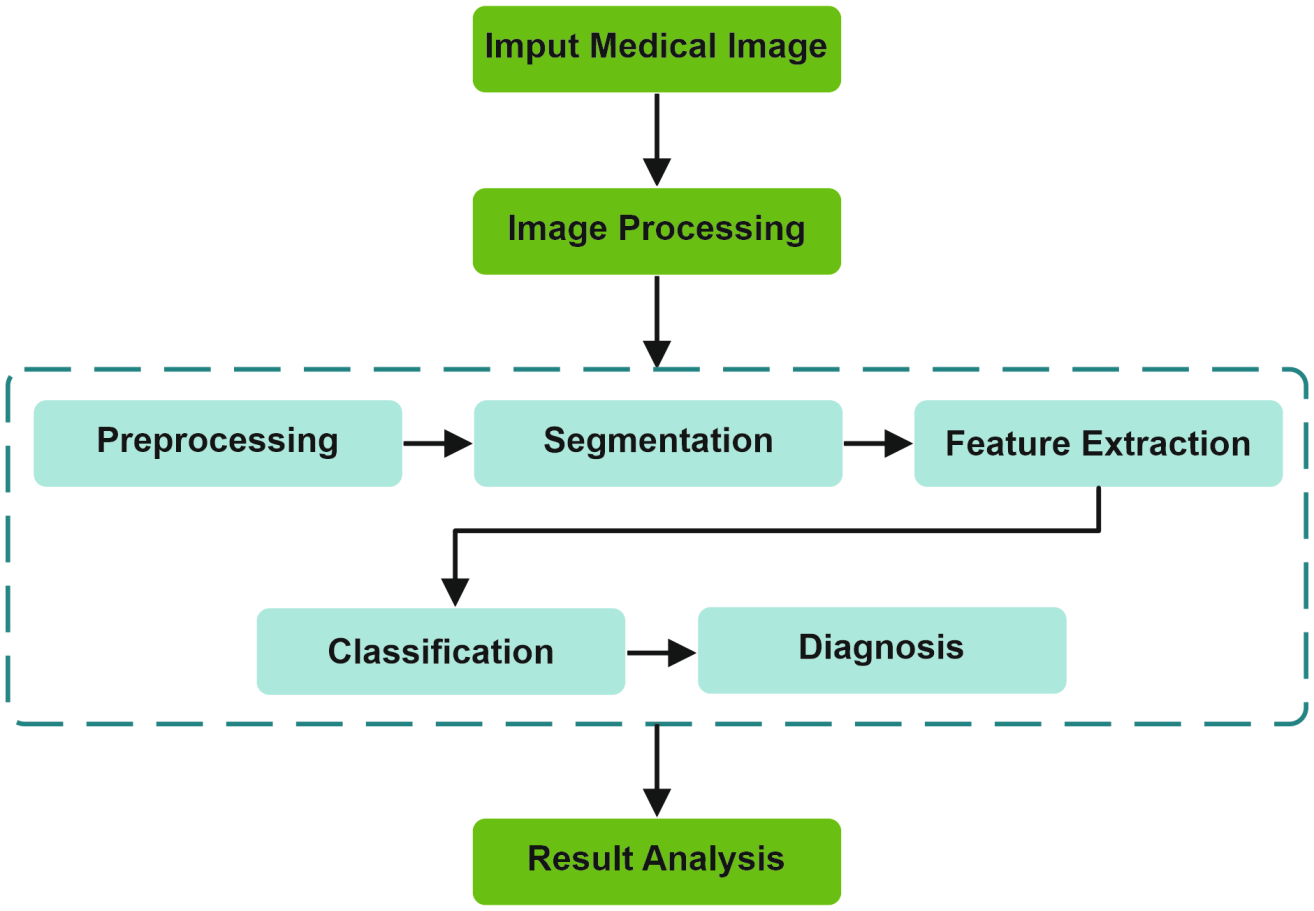
Stages of an AI algorithm.

The fundamental stages of an AI algorithm are illustrated in Figure 2. From the image acquisition to the last stage of result analysis, image processing is essential, undergoing multiple stages before reaching a diagnosis (22).

Over the past few years, there has been a rise in the number of publications exploring the role of AI in rheumatic diseases, reflecting the important current interest among rheumatologists in employing it for research (23).

**Figure 3:**
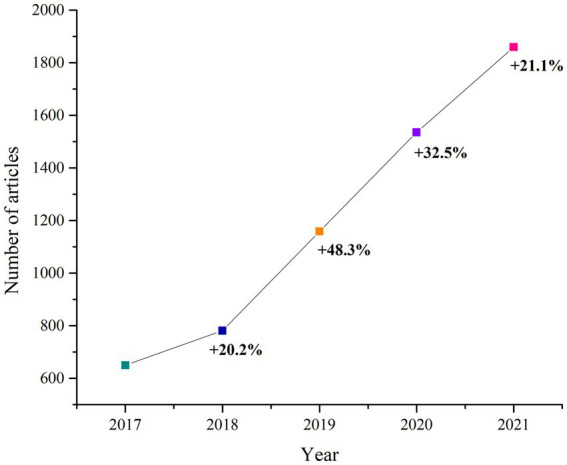
Evolution of publication patterns in Pubmed regarding AI.

When searching (23) for papers published in Pubmed from 2017 to 2021, related to the involvement of AI in rheumatic diseases, one can observe the increase in publications in 2021 compared to 2017 (Figure 3).

While the adoption of AI models for assessing inflammatory changes in RA and axSpA through MRI is still in its early stages, there is a significant number of studies on AI based on radiographs, ultrasound and CT images that have been published over the last years.

It is important to mention that in recent years, there has been an increase in the number of medical AI algorithms approved by the United States Food and Drug Administration (FDA), especially in the field of radiology. Thus, one example is the approval of an AI model, aimed efficiently on the diagnosis of knee osteoarthritis (OA) by conventional radiography.

AI algorithms on hand radiographs, including CNN, have been created and used for diagnosing RA, with an accuracy up to 95% (24, 25). Numerous publications have mentioned different algorithms for identifying and grading bone erosions, on hand radiographic images (24, 26, 27).

In a recent study, it was found that severity scores obtained through a DL-based model analyzing hand radiographs closely matched those assigned by the human eye (24, 28). Furthermore, AI models have also demonstrated effectiveness in detecting narrowing of joint spaces on radiographs (24, 27, 29).

Different AI models have been designed to assess synovitis through musculoskeletal ultrasound. Wu et al. created an algorithm incorporating DL to evaluate the severity of RA by classifying synovial proliferation observed through ultrasound (24, 30).

Moreover, studies focused on the use AI on CT images for automated detection and quantification of bone erosions suggest that this could enhance the precision of disease progression assessment when compared to traditional radiography (24, 31, 32).

However, rheumatic diseases have always represented a challenge for physicians, through their potentially disabling nature in which a prompt diagnosis is essential. MRI represent one of the most sensitive imaging methods for detecting inflammatory and structural changes occurring in non-radiographic axSpA, radiographic spondyloarthritis (SA) – formerly known as ankylosing spondylitis (AS), RA, OA and other rheumatic diseases. MRI allows detailed visualization of soft tissues, such as tendons, ligaments and joint capsule, as well as bone structures, compared to other imaging methods. However, MRI has certain limitations such as high cost, prolonged examination time, and the fact that interpretation depends on the experience of the radiologist. Thus, with the help of DL, sequence acquisition time can be reduced, acquisition protocols and interpretation could be improved, all with the goal of swiftly obtaining high quality assessments (12, 33).

Our purpose is to highlight the current state of AI research in MRI for the detection of inflammatory changes in RA and axSpA and to understand the potential role of implementing these techniques in clinical practice. Thus, the integration of AI in imaging has the potential to significantly improve diagnostic accuracy, efficiency, and patient outcomes.

## 2 Magnetic resonance imaging features

MRI is a non-invasive and non-irradiating method that allows a comprehensive multiplanar tomographic examination of joints, highlighting the bone structure, articular cartilage, synovial membrane, periarticular structures (ligaments, tendons) but also fluid accumulations (34).

T1-Weighted (T1W) sequences are very useful because they have a short acquisition time, provide concise anatomical details (bone erosions have low signal on T1W images) and can also highlight inflammation after the administration of the contrast agent (35). On the TW sequences, the fat tissue and the soft tissue highlighted by the administration of the contrast substance displays a strong signal. Because gadolinium (Gd) uptake is influenced by tissue vascularity and perfusion, the inflamed synovium, which is highly vascularized and perfused, becomes clearly visible. In T1W with fat saturation (FS) postcontrast acquisitions, FS allows the differentiation between the inflamed synovial membrane and the adjacent tissues (35, 36), thus making possible the easy recognition of the inflammatory process (35).

**Figure 4:**
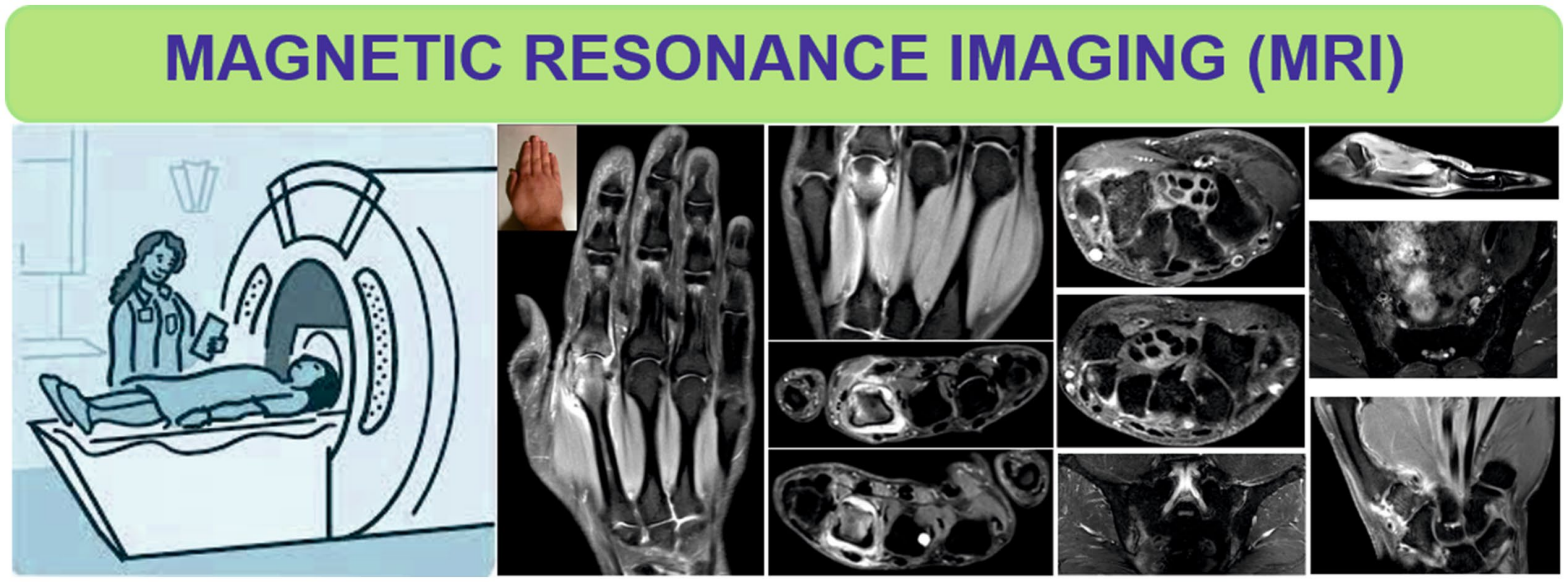
Magnetic resonance imaging of the hands and sacroiliac joints in patients with rheumatoid arthritis and axial spondyloarthritis.

T2-Weighted (T2W) sequences allow the visualization of features such as BME, fluid (Figure 4) as well as fat tissue, while T2W FS sequence is very useful in differentiating BME from lipomatous degeneration of bone structures (35, 36).

Another sequence that uses FS is the Short Tau Inversion Recovery (STIR) sequence, which allows a uniform and global suppression of the fat tissue, but which is not specific for its identification, the signal intensity of the tissues being similar in the short T1W and long T1W sequences.

Other sequences with affinity for fluid identification are proton density (PD) sequences with FS, T2 FS (saturation technique is the same in both sequences), and inversion recovery (IR) sequences (STIR/Turbo Inversion Recovery Magnitude (TIRM) - a different modality from FS). T2 and IR sequences are highly sensitive to fluids, while PD provides a higher signal-to-noise ratio (SNR) (35).

To improve the specificity of MRI diagnosis, post-contrast T1WFS can also be applied to detect synovitis or tenosynovitis and exudate, these not being necessary to identify BME and erosions (35, 37).

### 2.1 Rheumatoid arthritis

In 1998, a committee within the Outcome Measures in Rheumatology (OMERACT) group, involved in MRI research of inflammatory arthritis, began to work with the aim of integrating the MRI changes appearing in RA, into clinical trials. Currently there is an MRI score that is used in RA - Rheumatoid Arthritis Magnetic Resonance Imaging Scoring (RAMRIS) that was developed in 2000 (34, 38). It involves evaluation of subchondral bone erosions, BME and synovitis. The detection of BME through MRI can predict the location of later development of bone erosions and is thus considered a precursor lesion of the latter. Synovitis, which is easily highlighted by MRI, is in an interdependent relationship with the histological appearance (34, 39–42).

The OMERACT task force involved in the MRI research of RA suggests a standard protocol of sequences, with the aim of highlighting the inflammatory and destructive changes occurring at the level of the joints, these being (38, 43):

- T1W sequences in two planes, before and after the intravenous contrast administration.
- T2W sequence with FS; if not possible, a STIR sequence.

High-frequency MRI (≥1.5 T) joint assessment using the RAMRIS system typically includes metacarpophalangeal (MCP) joints 1–5 and the most affected wrist (MCP 1 being a recent addition). For these measurements, the most accurate assessment is possible by intravenous administration of the contrast agent (34). Synovitis and joint space narrowing are some of the main imaging features in RA which can be observed through MRI and have also been validated as outcomes measures (34, 44).

The RAMRIS system involves the assessment of both inflammatory features (such as synovitis and BME) and destructive lesions (such as erosions and joint space narrowing) that occur in the joints of patients with RA (34, 38, 45–47). Implementing this score in the imaging assessment allows for a more accurate monitoring of the disease over time.

The RAMRIS score is calculated as follows:

- a value from 0 to 3 is assigned for synovial inflammation, where 0 means the absence of any change and 3 represents severe damage;
- the presence of BME is evaluated from 0 to 3 depending on the volume of the affected bone (0-no edema, 1–1-33% edematous, 2–34-66% edematous and 3–67-100% edematous);
- bone erosions are scored from 0 to 10, depending on the affected bone surface (0 - no erosions, 1–1-10% of eroded bone, 2–11-20%, etc.).

With regard to bone erosions and edema, the RAMRIS score includes 23 areas at the level of each hand, each modified area receiving a score from 0 to 10 for erosions, respectively 0 to 3 for edema, depending of the degree of damage. In the case of synovitis, the distal radio-ulnar joints, the radio-carpal and intercarpal joints, as well as the II-V MCP joints are noted with a score from 0 to 3.

The standard imaging technique used in RA clinical trials is the RAMRIS score (1). Through MRI acquisitions, it has been shown in randomized controlled trials that there are significant differences between the efficacy of DMARDs at less than 6 months and even at less than 3 months, even in small groups of patients (1, 48–50). It has been shown that to identify differences in disease progression between two treatment groups in patients with early arthritis, plain radiography of the forefeet, both hands and wrists using Sharp/van der Heijde score requires twice the number of patients and twice the follow-up time compared to MRI performed at the MCP joints and the wrist of one hand (1, 48).

### 2.2 Axial spondyloarthritis

A definition of the active inflammatory changes occurring at the level of the SIJ, namely sacroiliitis, highlighted by MRI with the aim of classifying axSpA, was elaborated by the Assessment of SpondyloArthritis international Society (ASAS) in 2009. New methods of characterization of axSpA that include the notion of “ASAS-positive MRI” appeared in the same year. Currently, the notion of ASAS MRI has been widely adopted for the interpretation of the images obtained by MRI of the sacroiliac joints in everyday practice (4).

A diagnosis of axSpA is usually established in younger patients, which tipically manifest as chronic lower back pain with onset before 45 years of age. The ASAS classification criteria includes multiple other disease features (positive HLA-B27, uveitis, arthritis, increased CRP, enthesitis, dactylitis, psoriasis, etc.) in addition to imaging confirmation through radiography or MRI (for active sacroiliitis).

In the past 10 years, MRI studies of the spine and SIJ in patients with axSpA have significantly increased our understanding of the disease’s progression, it enabled early diagnosis and served as an objective endpoint for therapeutic trials. The correct technique for examining the SIJ involves sections in an oblique coronal plane at the level of the sacrum, using T1W turbo spin-echo (TSE), T2W gradient-echo (GRE) sequences, with in-phase (IP) and out-of-phase (OOP) sequences, as well as the STIR sequence with 4 mm thick slices. At least 10–12 slices are needed to cover the entire surface of the sacrum, from anterior to posterior. The administration of the contrast substance helps to identify inflammatory changes and should be followed by T1WFS sequences to better differentiate inflamed tissues from lipomatous degeneration (51).

The inflammatory changes that appear at the level of the SIJ, highlighted by MRI, can be presented in the form of enthesitis, capsulitis or BME (Figure 4), the latter being visualized adjacent to the joint surface, hyperintense in T2WFS sequences or in postcontrast enhancement T1W sequences (4, 52, 53).

The main MRI changes in SIJ that develop in axSpA are: BME (acute inflammatory lesion), lipomatous degeneration (chronic lesions that replace areas of BME), backfill (fat metaplasia within an erosion cavity) and the neoformation of bone tissue, eventually leading to ankylosis (4, 54–56).

According to ASAS, MRI scans suggestive of sacroiliitis should include presence of subchondral BME in at least two consecutive slices or in at least two different locations in a single slice. This was defined in order to exclude other structural or inflammatory changes such as enthesitis or capsulitis (4, 57, 58).

## 3 Applications of artificial intelligence based on MRI in rheumatoid arthritis

**Figure 5:**
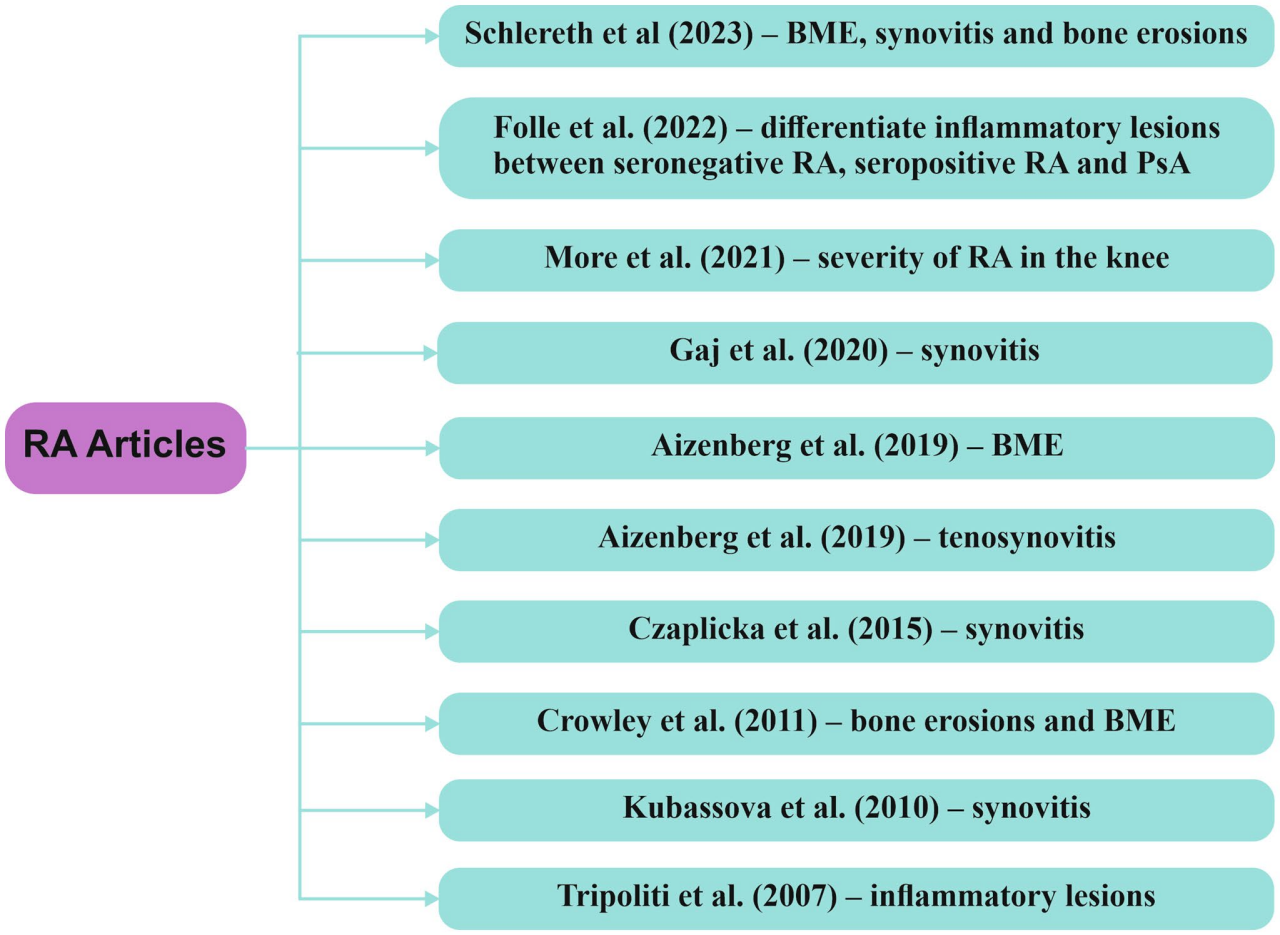
Research articles exploring computer-based techniques, with a focus on AI, in the analysis of MRI data within RA RA/rheumatoid arthritis, BME/bone marrow edema, PsA/psoriatic arthritis.

At this moment, the prevalent approach for the preclinical diagnosis of RA involves assessing ACPA and RF, yet the sensitivity of detecting these biomarkers is not notably high (24, 59, 60). The advancement of AI, ML and DL has resulted in a plethora of studies showcasing the potential of AI to aid in the early diagnosis, disease activity monitoring, and management of RA, through the analysis of clinical data, imaging, and laboratory samples. Figure 5 highlights studies which described the performance of various AI models implemented in the assessment of inflammatory changes through MRI in patients with RA.

Tripoliti et al. (61) devised a method for measuring and segmenting inflammatory changes in the hands using MRI T1W contrast sequences, involving 25 patients previously diagnosed with RA. The study demonstrated a sensitivity of 97,71% and a positive prediction rate of 83,35%. However, the number of false positive results was quite high, due to the identification of objects in the tendon sheaths that cannot be entirely eliminated, which suggests further improvement to eliminate false positive results.

Schlereth et al. (62) showcased the feasibility of automatically segmenting bone erosions, edema, and synovitis in the hands of 60 RA patients undergoing a 48-week treatment with the janus kinase inhibitor baricitinib. The segmentation was achieved using a 3D residual network (ResNet-3D), and assessments were conducted through the RAMRIS score and pre- and post-contrast T1W and T2W coronal MRI sequences. Noteworthy is the correlation between higher percentages of the area under the receiver operating characteristic curve (AUROC) and the area under the precision-recall curve (PR-AUC) with increased accuracy of the AI model. Throughout the treatment, a reduction in the RAMRIS score was observed, decreasing from 20,6 at week 0 to 18,3 at week 48. The AUROC results for bone erosions were 86 ± 2%, and PR-AUC was 83 ± 4%, while for edema, they were 78 ± 14 and 83% ± 10%, respectively. For synovitis, the AUROC was 60 ± 4%, and PR-AUC was 69 ± 3%, considering a smaller number of regions of interest (ROI). These findings suggest that the neural network model holds promise for potential future applications in clinical practice, offering high precision.

More et al. (22) introduced a DL model named ResNet50 for assessing the severity of RA in the knee, relying on the Kelgren Lawrence (KL) classification system, using MRI T1W, T2W and PD sequences. The study demonstrated a high level of accuracy at 96,85%, precision of 98,31%, area under curve (AUC) of 0,98 and a mean absolute error (MAE) of 0,015.

Aizenberg et al. (63) studied the feasibility of an automated approach for quantifying BME in the wrists of patients with early RA using MRI. The MRI images underwent processing through three distinct stages. It was determined that the automated technique for quantifying BME serves as a highly effective alternative to the visual assessment of BME. The correlation between the two methods—automatic and visual—proved to be robust, with a high Pearson correlation coefficient (p) (*r* = 0,83, *p* < 0,001).

In another study, Aizenberg (64) explored the feasibility of a method for quantifying tenosynovitis at the wrist through MRI in individuals with early RA. The model achieved very high correlation with visual assessment score of tenosynovitis (*r* = 0,93, *p* < 0,001). However, in contrast to the prior study, this one yielded false positive results because blood vessels and synovitis within ROI were frequently misidentified as tenosynovitis during quantitative measurements.

Kubassova et al. (65) introduced an automated model, Dynamika-RA, which significantly improved the quality of data by eliminating motion artifacts and reducing the time needed for MRI evaluation of synovitis in patients with RA. This advancement suggests the feasibility of incorporating this automated technique into clinical settings, with a focus on estimating disease progression and evaluating therapeutic efficacy.

A different automated model, created by Czaplicka et al. (66), focused on evaluating synovitis through MRI utilizing pre- and post-contrast T1W sequences in RA patients. The study revealed a robust correlation between the automatically measured volume and the manually calculated synovitis volume, along with the RAMRIS scores.

Gaj et al. (67) developed an automatic segmentation algorithm concentrating on detecting synovitis lesions in the wrists of RA patients using conditional generative adversarial networks and a convolutional network for image segmentation (U-Net). Despite being trained on a relatively modest dataset, this algorithm exhibited a reasonably satisfactory performance, achieving a Dice coefficient of 0,78.

The research conducted by Crowley et al. (68) assessed the viability, reability and feasibility of a computer-aided manual segmentation model in RA patients, comparing it with the RAMRIS scoring system. While the segmentation method demonstrated comparable consistency in quantifying erosions (intraclass correlation coefficient - ICC = 0,80) and high intraobserver reliability for both erosions (ICC = 0,994) and edema (ICC = 0,996), it exhibited lower interobserver reliability for BME (ICC = 0,46) and required a more extended timeframe (1–1,5 h for a single patient). In essence, the findings suggest that although the method may find application in clinical trials, significant challenges persist in terms of time and interobserver feasibility.

Further investigations have suggested the feasibility of integrating clinical and imaging data into a neural network capable of identifying inflammatory changes in RA. The ResNet neural network, as developed by Folle et al. (69), showcased the ability to differentiate among seronegative RA, seropositive RA, and psoriatic arthritis (PsA) based on inflammatory changes observed in MRI. The AUROC results indicated an accuracy of 75% in distinguishing seropositive RA vs. PsA, 74% for seronegative RA vs. PsA, and 67% for seropositive RA vs. seronegative RA. Notably, neural networks predominantly classified individuals with psoriasis as having PsA, implying the potential existence of a similar MRI pattern associated with PsA in the early stages of psoriatic disease.

## 4 Applications of artificial intelligence based on MRI in axial spondyloarthritis

The timely identification of axSpA through the correlation of clinical, imaging, and laboratory data holds immense significance for both healthcare professionals and patients. While radiography was the preferred method for evaluating SIJ many years ago, contemporary rheumatologists opt for MRI as the primary imaging choice. This shift is attributed to the MRI’s capability to detect early axSpA changes, surpassing the limitations of radiography.

**Figure 6:**
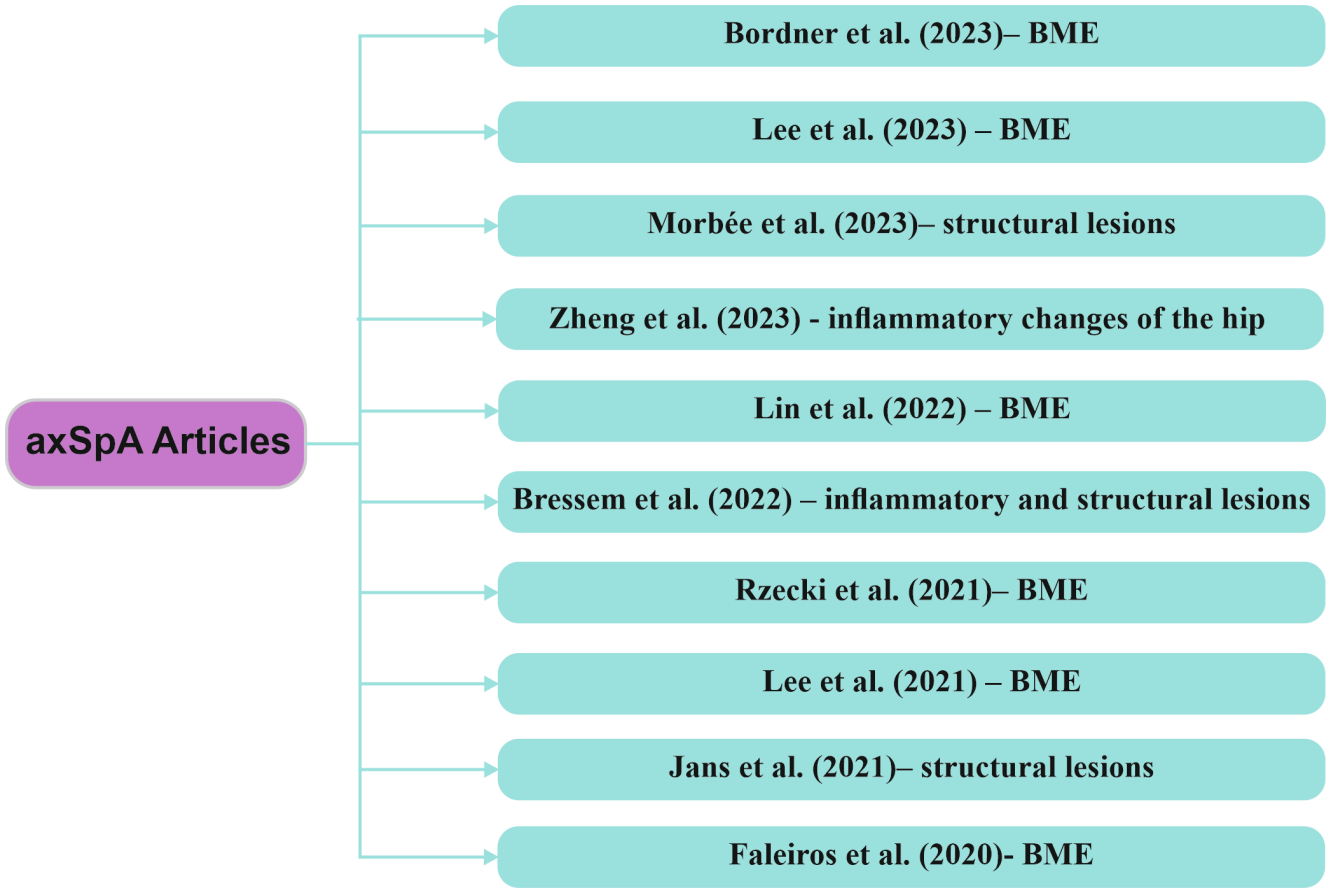
Research articles exploring computer-based techniques, with a focus on AI, in the analysis of MRI data within axSpA axSpA/axial spondyloarthritis, BME/bone marrow edema.

Research in the field of AI-based MRI assessment emphasizes the value of integrating models designed to recognize inflammatory changes associated with axSpA. These algorithms play a crucial role in achieving significant diagnostic outcomes, aiding not only in disease activity monitoring but also in disease management. Figure 6 highlights recent studies describing various AI models implemented in the assessment of inflammatory changes through MRI in patients with axSpA.

Rzecki et al. (70) developed a DL-based automatic segmentation algorithm for identifying BME through MRI examinations of the SIJ in patients diagnosed with axSpA. Following the validation of automatic inflammatory change assessments through manual evaluations, sensitivity increased from 0,88 to 0,95, specificity from 0,91 to 0,96, the Spearman correlation coefficient reached 0,866 between manually and automatically calculated lesion volumes, and the ICC was 0,9477. These findings confirm that automatic detection of inflammatory lesions is feasible, demonstrating high precision in assessing lesion volume.

Bordner et al. (71) created a DL model, named region-based convolutional neural network (mask-RCNN), designed to detect BME and predict the presence of active sacroiliitis on MRI based on the ASAS criteria (requiring BME to be identified in at least two different locations in a single slice). The model’s diagnostic efficacy in predicting active sacroiliitis according to ASAS criteria was assessed using sensitivity, specificity, Matthews correlation coefficient (MCC), accuracy and AUC. The study concluded that the model yielded results closely comparable to those of expert evaluations for both BME detection in the SIJ and the identification of active sacroiliitis according to ASAS criteria.

In another study Lee et al. (72) developed a DL model known as ResNet18-based network, using coronal oblique T1WFS contrast-enhanced (CE) MRI sequences of the SIJ as input data for BME detection in patients with axSpA. The study achieved an accuracy of 96,06% ± 2,83%, precision of 94,84% ± 3,73% and a recall rate of 100%. The results suggest that this study’s approach could serve as a valuable tool in clinical practice.

In a study, Lin et al. (73) introduced a DL model for the detection of BME utilizing STIR MRI sequences of the SIJ. The model’s results were compared with evaluations by a radiologist and a rheumatologist, exhibiting sensitivity and specificity similar with the radiologist’s assessment but surpassing that of the rheumatologist. Similarly, Faleiros et al. (74) utilized ML techniques to demonstrate the capability of identifying BME in patients with axSpA using MRI STIR sequences with promising results.

A retrospective study conducted by Lee et al. (75) involved the creation of two DL models: the R-CNN network for identifying SIJ by extracting ROI from MRI STIR sequences and the Visual Geometry Group-19 (VGG-19) network for detecting BME in patients with axSpA. The average AUROC accuracy was 0,898 and 0,830 at the image level and 0,801 and 0,827 at the patient level. These findings highlight the potential of DL techniques in diagnosing active sacroiliitis via MRI according to the ASAS criteria.

Zheng et al. (76) utilized a DL-driven MRI image assessment technique to detect inflammatory alterations in the hip among individuals with axSpA. The findings closely matched those of specialized radiologists, underscoring the substantial potential of this model to enhance diagnostic precision in axSpA patients, particularly emphasizing its relevance in clinical evaluations.

AxSpA diagnosis is based on the detection of both inflammatory changes and structural alterations (subchondral erosions, sclerosis, ankylosis) occurring at the SIJ. In the retrospective study conducted by Bressem et al. (77), a DL model was developed for the MRI assessment of inflammatory and structural lesions in SIJ in patients with axial axSpA. The results revealed a sensitivity of 88% and specificity of 71% for inflammatory changes, and a sensitivity of 85% and specificity of 78% for structural changes. The DL network achieved an AUC of 0,94, signifying a high level of performance.

Jans et al. (78) and Morbée et al. (79) conducted assessments of structural lesions using a synthetic CT, where the images obtained are generated from MRI sequences through a DL-based method. Their findings concluded that images obtained through synthetic CT demonstrated higher accuracy compared to T1W MRI sequences in detecting structural changes in patients with axSpA.

Ye et al. (80) developed and validated a nomogram model incorporating clinical risk factors and radiomic data to distinguish between axSpA and non-axSpA patients. The outcomes underscore the efficacy of this model, suggesting its potential implementation to enhance and simplify the process of making the best clinical decisions.

## 5 Discussion

The purpose of this review was to reveal the current state of AI integration in MRI for identifying inflammatory changes in RA and axSpA. The findings indicate that AI significantly contributes to the early detection of synovitis, BME, and bone erosions in these two rheumatic diseases, as supported by various studies (62, 63, 71, 75).

However, a constraint of this review is that it is focused only on the changes identified through MRI in two of the most common inflammatory joint pathologies, namely RA and axSpA. We mention that this research area was deliberately chosen because, in recent years, MRI has been on an upward trend regarding the early assessment of key changes in these two pathologies, surpassing radiography and CT.

Another limitation identified in our review is that the number of the studies to be reviewed was pretty narrow, but based on our research, there is a significantly greater number of published papers over the years using AI models based on ultrasonography, radiography and CT, compared to the number of published studies using MRI to assess changes in RA and axSpA.

**Figure 7:**
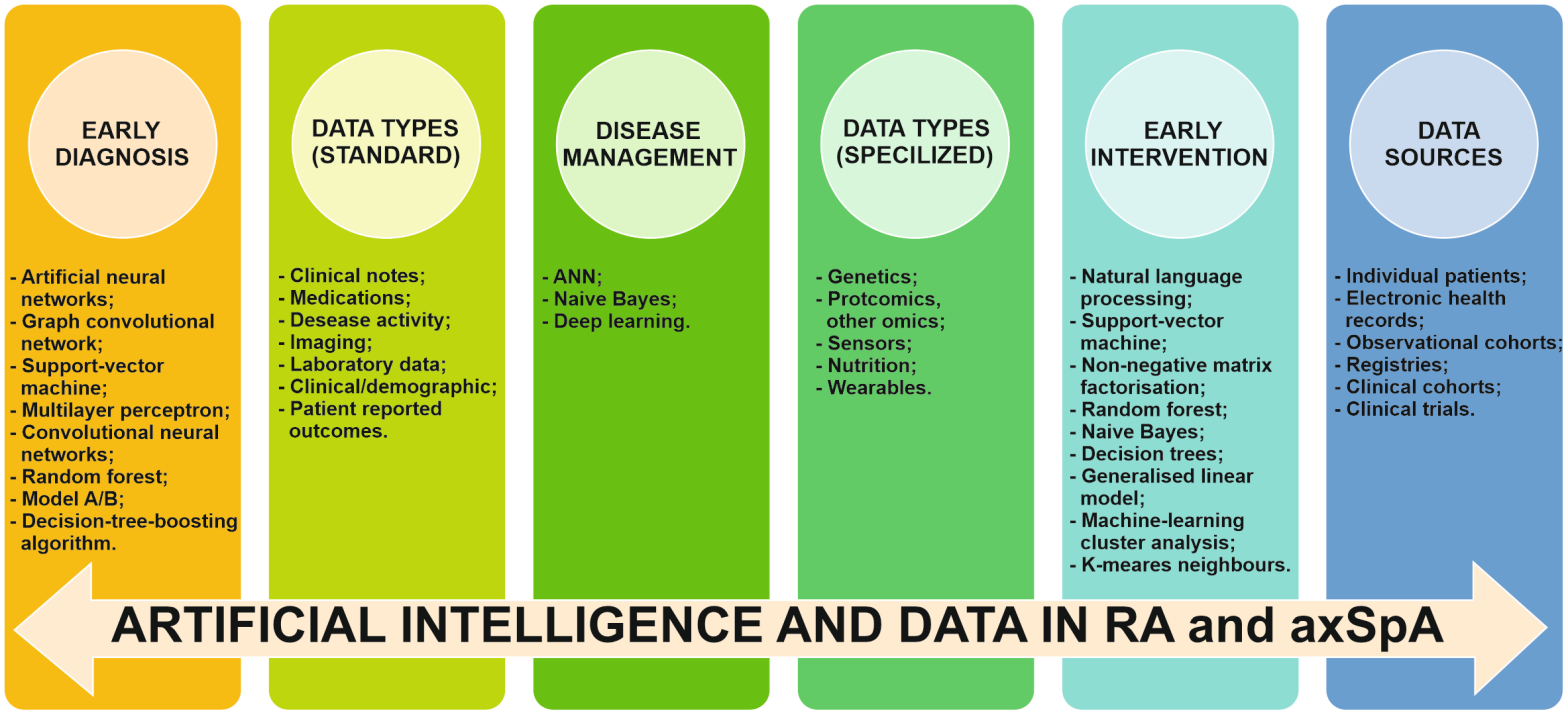
Implication of AI, data types and data sources in early diagnosis, early intervention and disease management of RA and axSpA RA/rheumatoid arthritis. axSpA/axial spondyloarthritis, ANN/artificial neural networks.

AI’s contribution has been demonstrated to enhance the accuracy of diagnosis in both RA and axSpA through the use of various datasets (Figure 7).

The increasing number of recent AI publications using MRI to assess RA and axSpA highlights the researchers’ interest for innovative methodologies, particularly the integration of neural networks with the aim to improve early diagnosis (81).

AI algorithms require thorough validation and testing using extensive and diverse datasets. This serves as a crucial measure to ensure their robustness and applicability across a broad spectrum of patient populations and clinical scenarios. Additionally, incorporating AI tools into existing clinical workflows poses distinctive challenges. These challenges encompass the creation of user-friendly interfaces, the implementation of comprehensive training programs for healthcare providers in AI usage, and the establishment of comprehensive guidelines for their application.

Adding to these challenges is the process of obtaining regulatory approval, a common obstacle when introducing any innovative medical technology. Furthermore, ethical considerations related to the use of AI, particularly concerning patient consent and data privacy, are yet to be thoroughly explored and resolved.

To navigate these complexities and bridge the gap between research and practice, fostering multidisciplinary collaborations is of paramount importance. Partnerships that involve AI researchers, clinicians, patients, policymakers, and regulatory bodies can facilitate the creation of expansive, representative datasets for algorithm training and validation. They can also assist in developing comprehensive guidelines for AI use in healthcare and creating strategies to effectively integrate AI into clinical practice.

The field of rheumatology stands to gain significantly from the integration of AI, and concerted efforts are expected to eventually overcome these challenges. This would lead to the incorporation of these state-of-the-art tools as a routine aspect of our clinical toolkit, fundamentally transforming our approach to diagnosing, classifying, and managing rheumatic diseases.

It is noteworthy in this context that the majority of the existing research on the application of MRI in rheumatology is retrospective. Therefore, there is a clear need for additional prospective studies. These studies would validate the use of MRI in clinical practice and offer a more comprehensive understanding of the potential and limitations of AI in this domain (12).

While recent studies on AI applications have shown promising results, specific constraints and challenges still hinder their seamless integration into clinical practice (18). AI methods require a diverse and extensive set of training data for both testing and validation. This is a fundamental step that AI techniques must undergo to attain viability and applicability across a broad spectrum of patients (72). AI faces a limitation in its proficiency for a specific task within a predefined context, restricting its ability to make decisions beyond the known task or context (73, 82–86). Essentially, AI is trained for a particular function and operates within a predetermined framework. For instance, AI algorithms are usually trained for a singular task, yet patients undergoing imaging examinations often exhibit multiple pathologies, requiring an algorithm trained for complex interpretation. Encountering difficulties in defining reference standards, such as inaccurate lesion segmentation or diagnostic uncertainties, can hinder the progress and limit the potential of an AI model.

Another factor that must not be ignored involves ethical considerations and the protection of personal data. There might be instances where patients decline participation in a study that incorporates an AI model due to a lack of information regarding the subsequent handling of their data.

A significant concern arises when an AI model contains a system error, as this could lead to severe consequences for a broad population if the model manages to proliferate (18).

While implementing AI algorithms in imaging, it is essential to take into account the mutual influence between the model’s accuracy and the quality of the input images. Therefore, as the quality of the obtained images improves, the accuracy of the AI model increases. Hence, AI specialists must be open to new approaches in exploring methods of visualizing and reconstructing images. These techniques need to be adapted to identify the specific characteristics of a condition with enhanced reproductibility. Therefore, it is important for experts to continuously adjust the AI algorithms to align with these emerging imaging methods or protocols (33).

Future advancements in AI models could enhance the accurate identification of changes in MRI sequences, potentially enabling the diagnosis of specific rheumatic diseases based on subtle characteristics alone. There is also potential for integrating MRI data with clinical, laboratory and genetic information to improve diagnostic accuracy. Training healthcare professionals, particularly radiologists and rheumatologists, to effectively use and interpret AI models implemented in MRI is crucial for their integration into clinical practice. Last but not least, long-term follow-up of patients involved in trials can provide valuable information about the progression of the disease, allowing for more precise monitoring and early treatment (72).

## 6 Conclusion

MRI is the most effective imaging method to detect inflammatory and structural changes, invisible to classic radiography. Through the integration of AI algorithms into MRI, it becomes feasible to achieve early identification of inflammatory changes, assess therapeutic efficacy, and monitor the activity of RA and axSpA.

In recent studies, the objective of developing and implementing AI in medical imaging follows a similar pattern. At times, the need for automation arises as an alternative to visual scoring due to its potential for reduced cost, time efficiency, and subjectivity. This can lead to decreased interobserver and intraobserver variability, requiring less extensive training and specific examiner skills. This objective is particularly pertinent in clinical trials, where the accurate detection of subtle changes or treatment effects is crucial. Additionally, automation is sought after for its potential to offer more specific measurements than visual scoring, as computer programs demonstrate greater consistency and are less prone to distraction by other image information.

AI exhibits significant potential in the field of rheumatology MRI, providing opportunities in disease diagnosis, classification, and management. By leveraging the capabilities of AI algorithms to scrutinize intricate imaging data like MRI, clinicians can enhance decision-making and formulate personalized treatment plans for individuals with rheumatic diseases. Nevertheless, challenges persist, including the necessity for extensive, high-quality datasets and the seamless integration of AI into clinical practice. Future research endeavors should prioritize overcoming these challenges and delving into novel applications of AI in rheumatology, aiming to enhance patient outcomes and revolutionize the field.

## Author contributions

A-IN: Conceptualization, Writing - original draft, Writing - review & editing. L-MS: Writing - original draft. CEB: Writing - original draft, Writing - review & editing. SCD: Conceptualization, Writing - review & editing. FAV: Conceptualization, Supervision, Writing - original draft, Writing - review & editing.

## Figures and Tables

**Table 1:** 

RA	rheumatoid arthritis
axSpA	axial spondyloarthritis
MRI	magnetic resonance imaging
AI	artificial intelligence
ML	machine learning
DL	deep learning
SIJ	sacroiliac joints
RF	rheumatoid factor
CRP	C-reactive protein
ACPAs	anti-cyclic citrullinated peptide antibody
ESR	erythrocyte sedimentation rate
T2T	treat-to-target
HLA-B27	leukocyte antigen B27
DMARDs	disease-modifying anti-rheumatic drugs
BME	bone marrow edema
CT	computed tomography
ANN	artificial neural networks
CNNs	Convolutional Neural Networks
FDA	United States Food and Drug Administration
OA	osteoarthritis
SA	spondyloarthritis
AS	ankylosing spondylitis
OMERACT	Outcome Measures in Rheumatology
RAMRIS	rheumatoid arthritis magnetic resonance imaging scoring
T1W	T1-weighted
T2W	T2-weighted
FS	fat saturation
STIR	Short Tau Inversion Recovery
MCP	metacarpophalangeal
Gd	gadolinium
IR	inversion recovery
PD	proton density
TIRM	Turbo Inversion Recovery Magnitude
SNR	single-to-noise ratio
ASAS	Assessment of SpondyloArthritis international Society
TSE	turbo-spin-echo
GRE	gradient-echo
IP	in-phase
OOP	out-of-phase
ResNet	residual network
AUROC	area under the receiver operating characteristic curve
PR-AUC	area under the precision-recall curve
ROI	region of interest
KL	Kelgren Lawrence
MAE	mean absolute error
p	Pearson correlation coefficient
ICC	intraclass correlation coefficient
PsA	psoriatic arthritis
mask-RCNN	region-based convolutional neural network
MCC	Matthews correlation coefficient
CE	contrast-enhanced
VGG	Visual Geometry Group
